# The Putative Role of Astaxanthin in Neuroinflammation Modulation: Mechanisms and Therapeutic Potential

**DOI:** 10.3389/fphar.2022.916653

**Published:** 2022-06-24

**Authors:** Shuai Wang, Xin Qi

**Affiliations:** Department of Physiology and Biophysics, Case Western Reserve University School of Medicine, Cleveland, OH, United States

**Keywords:** neuroinflammation, astaxanthin, medical application, antioxidation, neuroinflammatory factors, blood-brain barrier

## Abstract

Neuroinflammation is a protective mechanism against insults from exogenous pathogens and endogenous cellular debris and is essential for reestablishing homeostasis in the brain. However, excessive prolonged neuroinflammation inevitably leads to lesions and disease. The use of natural compounds targeting pathways involved in neuroinflammation remains a promising strategy for treating different neurological and neurodegenerative diseases. Astaxanthin, a natural xanthophyll carotenoid, is a well known antioxidant. Mounting evidence has revealed that astaxanthin is neuroprotective and has therapeutic potential by inhibiting neuroinflammation, however, its functional roles and underlying mechanisms in modulating neuroinflammation have not been systematically summarized. Hence, this review summarizes recent progress in this field and provides an update on the medical value of astaxanthin. Astaxanthin modulates neuroinflammation by alleviating oxidative stress, reducing the production of neuroinflammatory factors, inhibiting peripheral inflammation and maintaining the integrity of the blood-brain barrier. Mechanistically, astaxanthin scavenges radicals, triggers the Nrf2-induced activation of the antioxidant system, and suppresses the activation of the NF-κB and mitogen-activated protein kinase pathways. With its good biosafety and high bioavailability, astaxanthin has strong potential for modulating neuroinflammation, although some outstanding issues still require further investigation.

## Introduction

The initiation of neuroinflammation is physiologically responsible for phagocytosis and the clearance of cellular debris, aberrant proteins, and exogenous pathogens. This process is beneficial because it maintains the homeostatic environment and defends against exogenous insults in the brain. However, chronic or aberrantly prolonged inflammation can also cause devastating injury to resident cells of the central nervous system (CNS). Regulation of neuroinflammatory processes to maintain balanced innate immunity is crucial for brain homeostasis and intervening in CNS disorders (Marques-Deak et al., 2005; DiSabato et al., 2016; Fung et al., 2017).

Natural compounds with anti-inflammatory properties have sparked substantial interest as they can enhance neuroprotection. Carotenoids, a group of natural tetraterpenes that are the most abundant lipophilic pigments in nature, show great potential in medical applications (Milani et al., 2017; Sauer et al., 2019). They are found in various organisms, including plants, algae, bacteria, and fungi, and play vital roles in photosynthesis, photoprotection, anti-oxidation, biosynthesis of phytohormones, and signal transduction. Carotenoids are also crucial metabolic components and essential dietary supplements for animals with a deficiency in *de novo* carotenoid biosynthesis.

**FIGURE 1:**
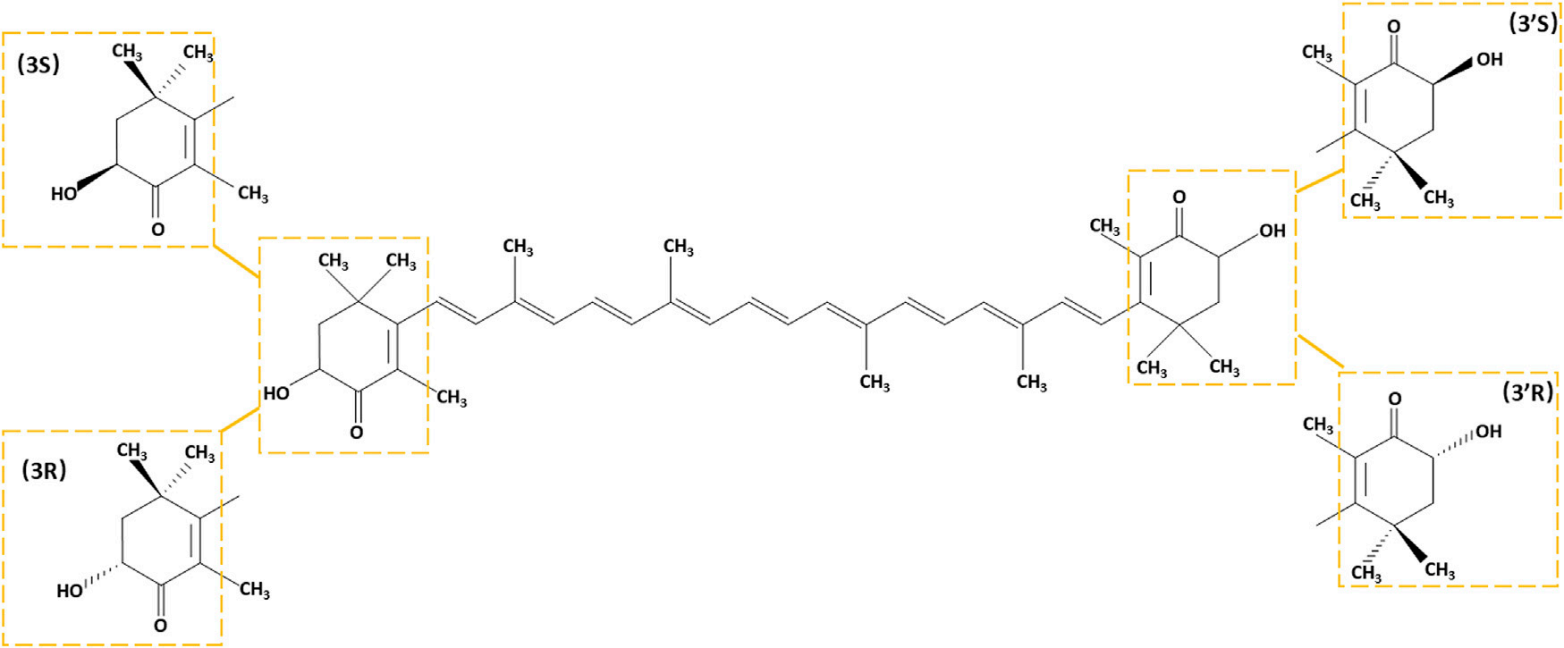
The chemical structure of astaxanthin. Stereoisomeric units are indicated with yellow boxes.

There are two types of carotenoids oxygen-free carotenes and oxygen-containing xanthophylls. Astaxanthin is one of the most common xanthophylls with an oxygen-containing group in its structure. Since its first isolation from a lobster in 1938 (Kuhn, 1938), astaxanthin has been used as a pigment and food additive for its good coloring properties. The fundamental structural feature of astaxanthin that resembles other carotenoids is a polyene chain comprising a battery of conjugated C=C bonds. Based on its molecular structure, astaxanthin is a 3,3′-dihydroxy-β,β′-carotene-4,4′-dione, containing two identical asymmetric carbon atoms at the 3 and 3′ positions of the β-ionone ring with a hydroxyl group at both ends. The 3,3′ asymmetric carbons allow astaxanthin to form three possible optical isomers with an all-trans configuration of the chain: 3R,3′R, 3S,3′S, and 3R,3′S (Figure 1). The ratios of astaxanthin stereoisomers vary widely in different organisms (Turujman et al., 1997; Osterlie et al., 1999; Coral-Hinostroza and Bjerkeng, 2002; Hussein et al., 2006), while synthetic astaxanthin is universally a racemic mixture composed of 25% 3R,3′R, 25% 3S,3′S, and 50% 3R,3′S isomers (Ambati et al., 2014). Astaxanthin can form monoesters and diesters, which is attributed to the reaction of its hydroxyl groups with fatty acids, such as palmitic, stearic, oleic, and linoleic acids. The esterified form generally dominates in different organisms, such as Antarctic krill, marine copepods and shrimps, and algae, while *Phaffia rhodozyma* predominantly contains its free form (Ambati et al., 2014).

Astaxanthin has been commercially developed for various applications in food ingredients, cosmetics, nutritional supplements, and pharmaceuticals due to its varied beneficial health effects that counter inflammatory, cancerous, diabetic, and cardiac diseases (Yuan et al., 2011). In recent years, an increasing number of studies have shown that astaxanthin can modulate neuroinflammation and be neuroprotective. In this review, we summarize the functional roles and mechanisms of action of astaxanthin in neuroinflammation and discuss the prospects and challenges for its potential therapeutic application in modulating neuroinflammation and protecting against neuroinflammation-associated disorders.

## Astaxanthin Modulates Neuroinflammation by Alleviating Oxidative Stress

### Oxidative Stress and Neuroinflammation

Neuroinflammation is generally recognized as an intriguingly complex process involving synergistic actions between neurons and different types of glial cells, including microglia, astrocytes, oligodendrocytes, and oligodendrocyte precursor cells. The coordinated interplay of these cells is mediated by neurotransmitters, ions, neurotrophic factors, and cytokines. Microglia are the most acute cells and usually the first to sense abnormalities in the brain microenvironment, even in their presumed resting state (Kreutzberg, 1996; Davalos et al., 2005; Nimmerjahn et al., 2005; Prinz et al., 2019). Acting as resident macrophages in the brain, microglia primarily play pivotal roles in initiating neuroinflammation. Under stress (e.g., local ischemia, mechanical injury, epilepsy, or exogenous pathogens) (Konat et al., 2006; Lehnardt, 2010; Fitzgerald and Kagan, 2020), injured neurons or oligodendrocytes can release neurotransmitters (i.e., ATP, glutamate, and nitric oxide) to activate microglia. In an inflammatory model, microglia were recruited to the injury site with the activation of intracellular inflammasomes and the production of pro-inflammatory cytokines (Liu GJ. et al., 2009; Duan et al., 2009; Dibaj et al., 2010; Gundersen et al., 2015; Song et al., 2021). Activated microglia can be broadly categorized into two subtypes, M1 and M2, which have pro- and anti-inflammatory roles, respectively. The traditional M1/M2 terminology for microglia was referenced from a classical macrophage polarization mode, which helped deduce different phenotypes of activated microglia in neuroinflammation processes. However, this biphasic partition appears to be an oversimplification as activated microglia also display mixed phenotypes and intermediate states (Hu et al., 2012; Nakagawa and Chiba, 2015; Orihuela et al., 2016; Ransohoff, 2016).

A dynamic redox equilibrium based on a balance between the production of reactive oxygen/nitrogen species (RONS) and the antioxidant defense system is crucial for maintaining normal cellular processes in the brain. Once excessive RONS overwhelm the defense system comprised of a series of antioxidant molecules and enzymes, oxidative stress occurs, with detrimental effects on various physiological processes. The brain is particularly susceptible to oxidative stress as elevated RONS can cause oxidative damage to brain resident cells, especially neurons and oligodendrocytes. A vast body of evidence shows that oxidative stress and neuroinflammation are inseparable and closely interrelated. Oxidative stress-induced neuronal damage or apoptosis promotes the release of neurotransmitters, such as ATP and nitrogen monoxide (NO), which trigger the initiation of neuroinflammation (Yang and Zhou, 2019). Moreover, reactive oxygen species (ROS) act as secondary messengers to evoke immune activation, while persistent inflammation can also facilitate oxidative stress (Simpson and Oliver, 2020). Reactive nitrogen species (RNS) can activate matrix metalloproteinases (MMPs) to trigger blood-brain barrier (BBB) disruption and neuroinflammation (Chen HS. et al., 2018; Hannocks et al., 2019). Consequently, the interplay of RONS generation and neuroinflammation leads to a vicious circle, resulting in persistent damage or degeneration of the brain (Dias et al., 2013; Agrawal and Jha, 2020; Teixeira-Santos et al., 2020; Tewari et al., 2021).

### Mechanisms by Which Astaxanthin Defends Against Oxidative Stress

Astaxanthin is a superior antioxidant for neutralizing RONS. ROS are defined as highly reactive oxidizing free radical agents, consisting of superoxide anions (O_2_•), hydroxyl (OH•), peroxyl (ROO•), and hydrogen peroxide (H_2_O_2_) radicals. RNS mostly consists of NO, nitrogen dioxide (NO_2_), and peroxynitrite (ONOO^−^). They all exhibit high reactivity to proteins, lipids, and DNA (Valko et al., 2006; Ryter et al., 2007; Valko et al., 2007). Therefore, the aberrant accumulation of RONS can lead to the impairment of cellular components associated with cellular senescence and various diseases (Gorrini et al., 2013; Sies, 2015; Bisht et al., 2017; van der Pol et al., 2019). Carotenoids have attracted considerable interest for their potent antioxidant activity. Several studies published almost 30 years ago revealed that the anti-oxidative activity of carotenoids is mediated by quenching singlet oxygen and free radicals (Palozza and Krinsky, 1992b; a; Tsuchiya et al., 1992). Astaxanthin has higher antioxidant activity by scavenging peroxyl radicals than other carotenoids, such as lycopene, β-carotene, α-carotene, and lutein (Naguib, 2000). It is about 550 times more capable of neutralizing singlet oxygen than α-tocopherol (Shimidzu et al., 2008). The powerful antioxidant capacity of astaxanthin depends both on the polyene system found in other carotenoids and on the terminal rings that are unique to its structure (Britton, 1995; Miller et al., 1996). Its polar β-ionone ring with a hydroxyl group at either end gives it a higher capacity to neutralize free radicals. It is postulated that astaxanthin in a dihydroxy-conjugated polyene form possesses a hydrogen atom suitable for blocking free radical reactions like that of α-tocopherol (Higuera-Ciapara et al., 2006).

In addition to direct radical scavenging, astaxanthin can also regulate the cellular enzymatic system to defend against excessive ROS production. Nuclear factor erythroid 2-related factor (Nrf2) is a pivotal transcription factor acting as the guardian of redox homeostasis and is considered a prospective therapeutic target for oxidative stress- and inflammation-associated diseases (Innamorato et al., 2008; Ahmed et al., 2017; Zhang R. et al., 2017). Nrf2 regulates the transcriptional activation of many cytoprotective genes, such as those encoding NADPH quinone dehydrogenase 1 (Nqo1), glutathione-S-transferase-α1 (GST-α1), and heme oxygenase-1 (H O -1), which protect against oxidative stress and inflammation (Telakowski-Hopkins et al., 1988; Rushmore et al., 1991; Friling et al., 1992; Lu et al., 2016). For instance, HO-1 catalyzes the degradation of heme into carbon monoxide, free iron, and biliverdin. Monoxide functions as an inhibitor of the nuclear factor-κB (NF-κB) pathway, contributing to the decreased expression of pro-inflammatory cytokines. It can also directly inhibit pro-inflammatory cytokines and activate anti-inflammatory cytokines, alleviating inflammation (Ahmed et al., 2017).

**FIGURE 2:**
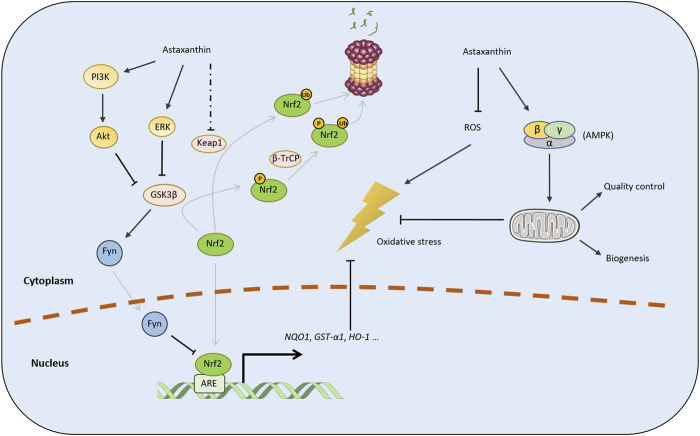
Mechanisms by which astaxanthin defends against oxidative stress. Astaxanthin mitigates oxidative stress by directly scavenging radicals and regulating the cellular antioxidative enzymatic system via the Nrf2 pathway. The black arrows represent positive regulation, while the lines with T-shaped ends represent inhibition. A dotted line indicates an inconclusive pathway. The gray lines indicate phosphorylation, ubiquitination, or nuclear translocation. P and Ub represent the phosphorylation and ubiquitination of target proteins, respectively.

Astaxanthin can activate the Nrf2 pathway by promoting the activity of phosphoinositol-3 kinase/protein kinase B (PI3K/Akt) and extracellular signal-regulated protein kinase (ERK) pathways (Wang et al., 2010; Li et al., 2013). PI3K/Akt and ERK can promote the nuclear translocation of Nrf2, although the underlying mechanism has not been completely elucidated. Several E3 ligase adaptor proteins, such as Kelch-like ECH-associated protein 1 (Keap1) (Nguyen et al., 2003; Stewart et al., 2003), β-transducing repeat-containing protein (β-TrCP) (Rada et al., 2011; Cuadrado, 2015), and synoviolin 1 (Hrd1), tightly regulate Nrf2 levels (Wu et al., 2014). Some studies and reviews have suggested that astaxanthin may inhibit Nrf2 degradation via the Keap1 pathway (Wu et al., 2015; Fakhri et al., 2019). However, whether astaxanthin regulates Keap1 expression remains unclear as different studies presented controversial conclusions (Li L. et al., 2020; Ma et al., 2020). Although astaxanthin can promote ERK activity (Wang et al., 2010), disruption of the Keap1 from Nrf2 is not dependent on ERK activation, suggesting that astaxanthin activates Nrf2 via another pathway (Zipper and Mulcahy, 2003). The phosphorylation of Nrf2 mediated by glycogen synthase kinase 3β (GSK3β) can facilitate its ubiquitination and proteasomal degradation via β-TrCP (Cuadrado, 2015; Mathur et al., 2018). Moreover, GSK3β can activate the Fyn tyrosine kinase to induce the nuclear export of Nrf2 for its ubiquitination and degradation (Jain and Jaiswal, 2007; Niture et al., 2014). Considering that PI3K/Akt and ERK inhibit the activity of GSK3β (Ding et al., 2005; Kaidanovich-Beilin and Woodgett, 2011; Manning and Toker, 2017), we speculate that astaxanthin enhances the stability of Nrf2 by inactivating the GSK3β/β-TrCP or GSK3β/Fyn pathway (Figure 2).

Astaxanthin can also regulate mitochondrial function in response to oxidation stress (Kim and Kim, 2018). Astaxanthin pretreatment can restored mitochondrial membrane potential (MMP) and significantly inhibit hydrogen peroxide-induced apoptosis of primary cortical neurons (Lu et al., 2010). Similarly, astaxanthin can improve mitochondrial function in a reduced state under oxidative stress (Wolf et al., 2010). Some evidence indicates that astaxanthin contributes to mitochondrial quality control and promotes mitochondrial biogenesis through the AMP-activated protein kinase (AMPK) pathway, however, the underlying mechanism remains unclear (Nishida et al., 2020; Nishida et al., 2021).

### Neuroprotective Effect of Astaxanthin is Mediated by Inhibiting Oxidative Stress

Astaxanthin inhibits neuroinflammation by alleviating oxidative stress, thereby exerting a beneficial neuroprotective effect. Oxidative stress is a major cause of neuronal damage-induced neuroinflammation. Astaxanthin protects against neuronal loss in the rat hippocampus caused by epilepsy by attenuating oxidative damage (Lu et al., 2015). Moreover, the treatment of rats with astaxanthin attenuated epilepticus-induced cognitive dysfunction by inhibiting oxidative stress and neuroinflammation and mitigating a decrease in Nrf2 levels (Deng et al., 2019). Similarly, astaxanthin can prevent lanthanum oxide nanoparticle-induced hippocampal injury by reducing oxidative stress and neuroinflammation via the PI3K/AKT/Nrf-2 pathway (Yuan et al., 2020). Furthermore, astaxanthin ameliorates lipopolysaccharide (LPS)-induced oxidative stress, neuroinflammation, and memory dysfunction (Han et al., 2019). Astaxanthin also significantly protects against doxorubicin-induced memory impairment by blocking oxidative, inflammatory, and pro-apoptotic insults (El-Agamy et al., 2018). The ROS accumulating during oxidative stress are crucial triggers of microglial polarization (Simpson and Oliver, 2020). Astaxanthin treatment can halt M1 and promote M2 microglial polarization in response to LPS, suppressing neuroinflammation in BV2 microglial cells (Wen et al., 2017; Zhou et al., 2021). Consistent with its inhibitory role against microglial activation, astaxanthin can suppress the release of ATP from microglia by reducing the P2X7 receptor levels, although the mechanism underlying this remains elusive (Wang M. et al., 2020).

## Astaxanthin Modulates Neuroinflammation by Inhibiting Pro-Inflammatory Cytokine Production

The aberrant production of pro-inflammatory cytokines in the CNS is a representative feature of neuroinflammation. Astaxanthin can inhibit the production of several pro-inflammatory cytokines, such as interleukin 1β (IL-1β), interleukin 6 (IL-6), and tumor necrosis factor-α (TNF-α), via repressing the NF-κB and mitogen-activated protein kinase (MAPK) pathways.

### NF-κB Pathway

The NF-κB transcription factor family, a prototypical mediator of inflammation, is crucial for innate and adaptive immune responses. NF-κB family members, namely RelA (p65), RelB, c-Rel, NF-κB1 (p50), and NF-κB2 (p52), form homo- or heterodimers that activate the transcription of target genes by binding to a specific DNA element (Wan and Lenardo, 2009). NF-κB pathways can be classified into canonical and non-canonical (alternative) signaling pathways, which are induced by different pro-inflammatory cytokines through the participation of different family members. In the canonical NF-κB pathway, diverse stimuli, (e.g., LPS, TNFα, and IL-1) can trigger the activation of the multi-subunit IκB kinase (IKK) complex that further phosphorylates IκBα at two N-terminal serines and induces ubiquitin-mediated IκBα degradation. In most quiescent conditions, NF-κB signaling is inactivated because the dimers, (e.g., p50/RelA and p50/c-Rel) are bound to the inhibitory protein, IκBα (Senftleben et al., 2001; Hayden and Ghosh, 2008; Liu et al., 2017). The induced degradation of IκBα alleviates this inhibition, resulting in the transient nuclear translocation of the NF-κB dimers and subsequent expression of various pro-inflammatory factors, including cytokines, chemokines, and adhesion molecules (Hoesel and Schmid, 2013). In contrast, the non-canonical NF-κB pathway involves the processing of the NF-κB2 precursor protein (p100) by TNF receptor (TNFR) superfamily receptors (Sun, 2012; 2017). NF-κB-inducing kinase and IKKα mediate p100 phosphorylation and processing into p52, which then induces the transcriptional activation of target genes by forming a heterodimer with RelB (Senftleben et al., 2001; Xiao et al., 2001). Functionally, both NF-κB pathways are important in regulating different aspects of the innate and adaptive immune responses (Sun, 2011; Liu et al., 2017; Sun, 2017).

**FIGURE 3:**
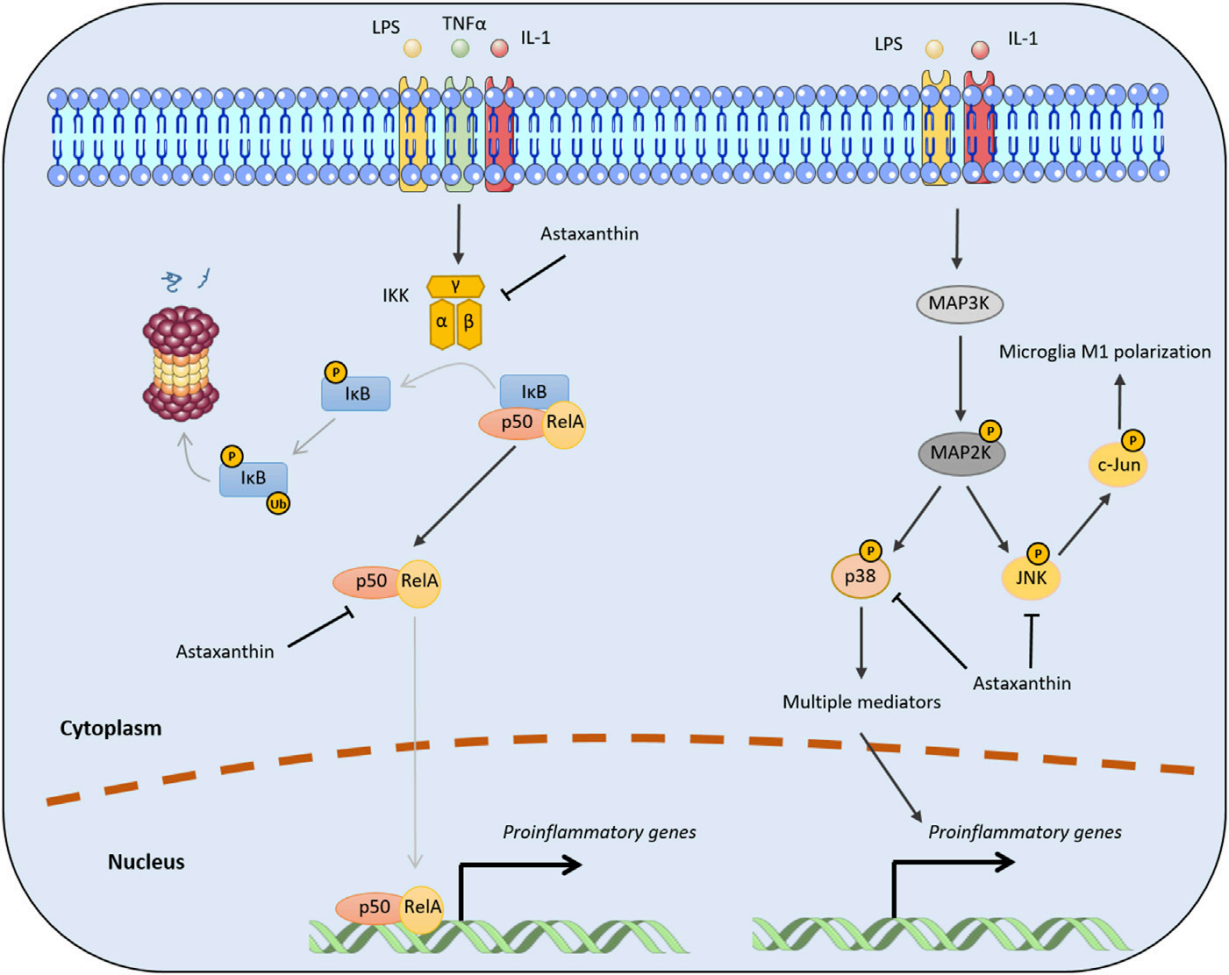
Molecular mechanisms by which astaxanthin inhibits pro-inflammatory cytokine production. The black arrows represent positive regulation, while the lines with T-shaped ends represent inhibition. Gray lines indicate the processes of phosphorylation, ubiquitination, or nuclear translocation. P and Ub represent the phosphorylation and ubiquitination of target proteins, respectively.

Mounting evidence indicates that astaxanthin inhibits neuroinflammation by halting NF-κB activation through the canonical NF-κB pathway. For example, administration of astaxanthin after the onset of status epilepticus in a rat model abrogated the induced expression of several inflammatory factors (e.g., cytochrome c oxidase subunit II [Cox-2], IL-1β, and TNFα) and p65 phosphorylation in the hippocampus and parahippocampal cortex (Deng et al., 2019). In addition, trans-astaxanthin could effectively antagonized LPS-induced TNF-α, IL-1β, and IL-6 expression in the hippocampus and the prefrontal cortex by regulating the NF-κB pathway (Jiang et al., 2016). Moreover, Zhang et al. (2014a) reported administration of a high dose of astaxanthin after subarachnoid hemorrhage significantly downregulated NF-κB DNA binding activity and the expression of inflammatory cytokines and intercellular adhesion molecule. Mechanistically, astaxanthin can effectively reduce NF-κB-related inflammation by suppressing IKKβ phosphorylation and the nuclear translocation of the p65 subunit (Bhuvaneswari et al., 2014). Moreover, astaxanthin can decrease p65 phosphorylation, which may impair the nuclear translocation and DNA binding activity of p50/p65 dimers (Figure 3) (Terazawa et al., 2012; Zhang et al., 2014a).

### MAPK Pathway

The MAPK family of serine/threonine kinases is involved in the immune response (Arthur and Ley, 2013). There are 14 known MAPK proteins in mammalian cells, that function in seven distinct signaling pathways (Mathien et al., 2021). Based on structural and functional features, MAPK family members can be divided into classic MAPKs, consisting of the ERK1/2, c-Jun N-terminal kinases (JNK1/2/3), p38 (p38a/b/c/d), and ERK5 subfamilies, and atypical MAPKs, which include the ERK3/4, ERK7, and Nemo-like kinase subfamilies (Coulombe and Meloche, 2007; Mathien et al., 2021). The MAPK signaling pathway involves a cascade of three kinases that lead to the successive phosphorylation of different kinase targets. Briefly, MAPK kinase (MAP3K) is first activated in response to pathogen infection or tissue damage through the Toll-like or interleukin-1 receptors. Accordingly, MAP3K activates a MAPK kinase (MAP2K), which activates MAPK subfamily members by dual phosphorylation of the Thr–X–Tyr activation motif. Finally, MAPK proteins promote inflammatory reactions by producing different pro-inflammatory cytokines (Dumitru et al., 2000) and modulating macrophage polarization (Rincon and Davis, 2009).

The mechanism by which astaxanthin affects MAPK signaling pathways is intrinsically linked to its regulation of MAPK proteins, which act as inflammatory signaling mediators. Consistent with this mechanism, a molecular docking study identified an interaction between astaxanthin and human p38. Astaxanthin inhibits p38 by occupying its active site and interacting with surrounding amino acid residues (Yang et al., 2019). Moreover, astaxanthin can promote M2 polarization of BV2 cells and suppress neuroinflammation by inhibiting NF-κB and JNK signaling (Figure 3). Specifically, astaxanthin can reduce phosphorylated c-Jun levels which is indicative of the inactivation of JNK signaling, although the mechanism underlying this remains unclear (Wen et al., 2017).

## Astaxanthin Modulates Neuroinflammation by Maintaining Blood–Brain Barrier Integrity and Inhibiting Peripheral Inflammation

### BBB, Peripheral inflammation, and Neuroinflammation in the CNS

Disruption of the BBB and peripheral inflammation are two factors contributing to neuroinflammation. The mammalian brain has an intricate system of blood vessels; cerebral blood vessels measure ∼640 km with an endothelial surface area of ∼12 m^2^ (Abbott et al., 2010) ensuring efficient molecular exchange. The BBB is formed by vascular endothelial cells, mural cells (e.g., pericytes and smooth muscle cells), and perivascular astrocytic end-feet around capillaries (or glial limitans ensheathing the penetrating arterioles (Yao et al., 2014)). Its permeability is also regulated by surrounding microglia and neurons (Zhao et al., 2015; Sweeney et al., 2019). The BBB can prevent blood cells, neurotoxic components, and pathogens from entering the brain, while its breakdown and dysfunction can lead to various neurological deficits (Obermeier et al., 2013; Liebner et al., 2018). Peripheral inflammation involves the activation of the immune system outside of the CNS and the release of pro-inflammatory cytokines against various pathological stimuli in the peripheral blood. Peripheral inflammation is a crucial trigger of BBB disruption because increasing the levels of pro-inflammatory cytokines and cytotoxic pathogens or molecules in the plasma during infections damages its integrity (Huang et al., 2021). Impaired BBB permeability allows peripheral inflammatory molecules and signals to access the brain, leading to neuroinflammation. Moreover, neuroinflammation can further contribute to BBB disruption by damaging endothelial tight junction proteins (Schreibelt et al., 2007). Leukocytes (e.g.,monocytes, neutrophils, and T- and B-lymphocytes) and secreted inflammatory cytokines in the peripheral blood can infiltrate the brain due to BBB dysfunction, triggering or exacerbating the progression of neuroinflammation (Kim et al., 2016; Gimenez-Arnau et al., 2021; Pluta et al., 2021). Therefore, improving BBB integrity and mitigating peripheral inflammation can be neuroprotective by inhibiting neuroinflammation.

### Astaxanthin Protects BBB integrity

**FIGURE 4:**
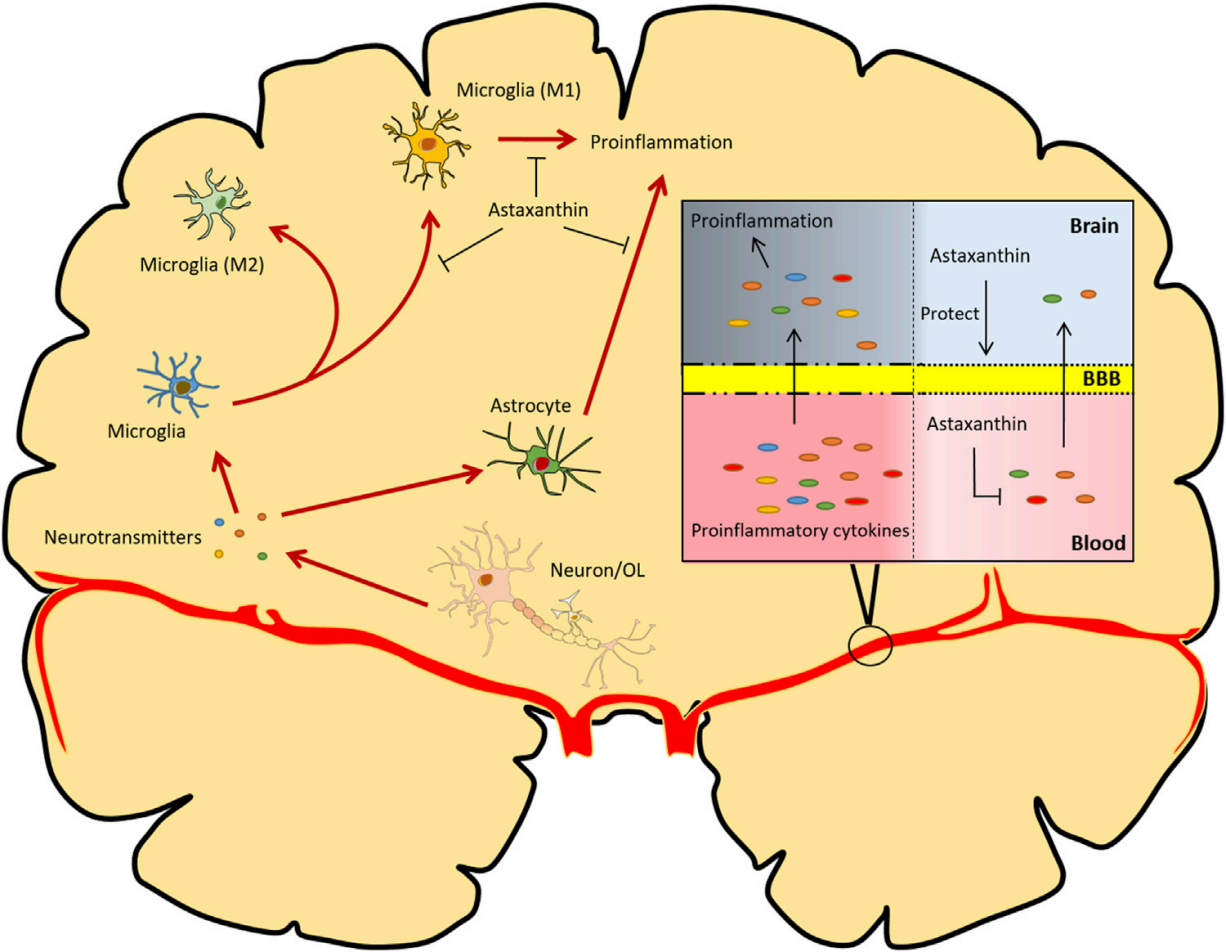
Overview of the functional implications of astaxanthin in modulating neuroinflammation. Astaxanthin counteracts oxidative stress-induced damage to neurons and glial cells. Astaxanthin can also reduce the production of pro-inflammatory cytokines in the brain and retard the M1 polarization of microglia. Additionally, astaxanthin protects BBB integrity and inhibits the production and infiltration of inflammatory cytokines derived from peripheral inflammation. OL, oligodendrocytes. The red arrows represent processes of neuroinflammation; the black arrows represent promotion, protection or transportation; the lines with T-shaped ends represent inhibition.

Astaxanthin has enormous potential for protecting the BBB from disruption or dysfunction. Astaxanthin treatment after subarachnoid hemorrhage significantly reduces brain edema, BBB dysfunction, and concomitant neuroinflammation in rat and rabbit models (Zhang et al., 2014a; Zhang et al., 2014c; Zhang et al., 2015). Similarly, pretreatment with astaxanthin prevents the BBB disruption and neuroinflammation caused by kaliotoxin (Sifi et al., 2016). Mechanistically, astaxanthin can restore the survival rate, increase oxidative stress resistance, and maintain the tight junction stability of rodent brain microvascular endothelial cells in response to oxygen-glucose deprivation/reperfusion treatment or subarachnoid hemorrhage, indicating its effectiveness in protecting the BBB (Zhang et al., 2015; Kuo et al., 2019) (Figure 4).

### Astaxanthin inhibits Peripheral inflammation

The anti-inflammatory properties of astaxanthin have been demonstrated in various *in vivo* and *in vitro* studies. For example, astaxanthin decreased mRNA and protein expression levels of pro-inflammatory genes in macrophages, including TNF-α, transforming growth factor (TGF-β), IL-1β, IL-6, COX-2, and inducible nitric oxide synthase (iNOS) (Kishimoto et al., 2010; Farruggia et al., 2018; Cai et al., 2019; Kang et al., 2020; Binatti et al., 2021). It could also inhibit the expression of pro-inflammatory cytokines in human corneal epithelial cells (Li H. et al., 2020) and keratinocytes (Terazawa et al., 2012). Similarly, astaxanthin could suppress the activation of the NOD-, LRR-, and pyrin domain-containing protein 3 (NLRP3) inflammasome in macrophages (Peng et al., 2020). Moreover, multiple *in vivo* studies have revealed the anti-inflammatory effects of astaxanthin in different disease models, including non-alcoholic fatty liver (Bhuvaneswari et al., 2014; Ni et al., 2015; Chiu et al., 2016; Jia et al., 2016), hepatic injury or fibrosis (Zhang J. et al., 2017; Han et al., 2018; Liu et al., 2018; Zhang Z. et al., 2020), kidney injury (Guo et al., 2021), myocardial injury (Xie et al., 2020), diabetes mellitus (Feng et al., 2020; Liu et al., 2020; Zhuge et al., 2021), arthritis (Park MH. et al., 2020; Kumar et al., 2020), gastroenteritis inflammation (Han et al., 2020; Chen Y. et al., 2021), acute pancreatitis (Yasui et al., 2011), asthma (Hwang et al., 2017), atopic dermatitis (Park et al., 2018; Park et al., 2019), and hyperosmoticity-induced dry eye (Li H. et al., 2020). As described earlier, astaxanthin counters inflammation primarily by blocking the NF-κB-dependent signaling pathways. However, astaxanthin can also promote cyclooxygenase inhibition and downregulation of prostaglandin and TNF-α by decreasing nitric oxide (NO) production and iNOS activity in an NF-κB pathway-independent manner (Ohgami et al., 2003). Additionally, dietary astaxanthin supplementation in females decreases DNA oxidative damage and lipid-peroxidation, reduces C-reactive protein concentrations, enhances natural killer cell cytotoxic activity, and increases total T- and B-cell subpopulations, indicating the beneficial effects of astaxanthin in ameliorating oxidative stress and inflammation and in improving the immune response, although the mechanisms involved require further elucidation (Park et al., 2010).

## Therapeutic Benefits of Astaxanthin in Neuroinflammation-Associated Disorders

**FIGURE 5:**
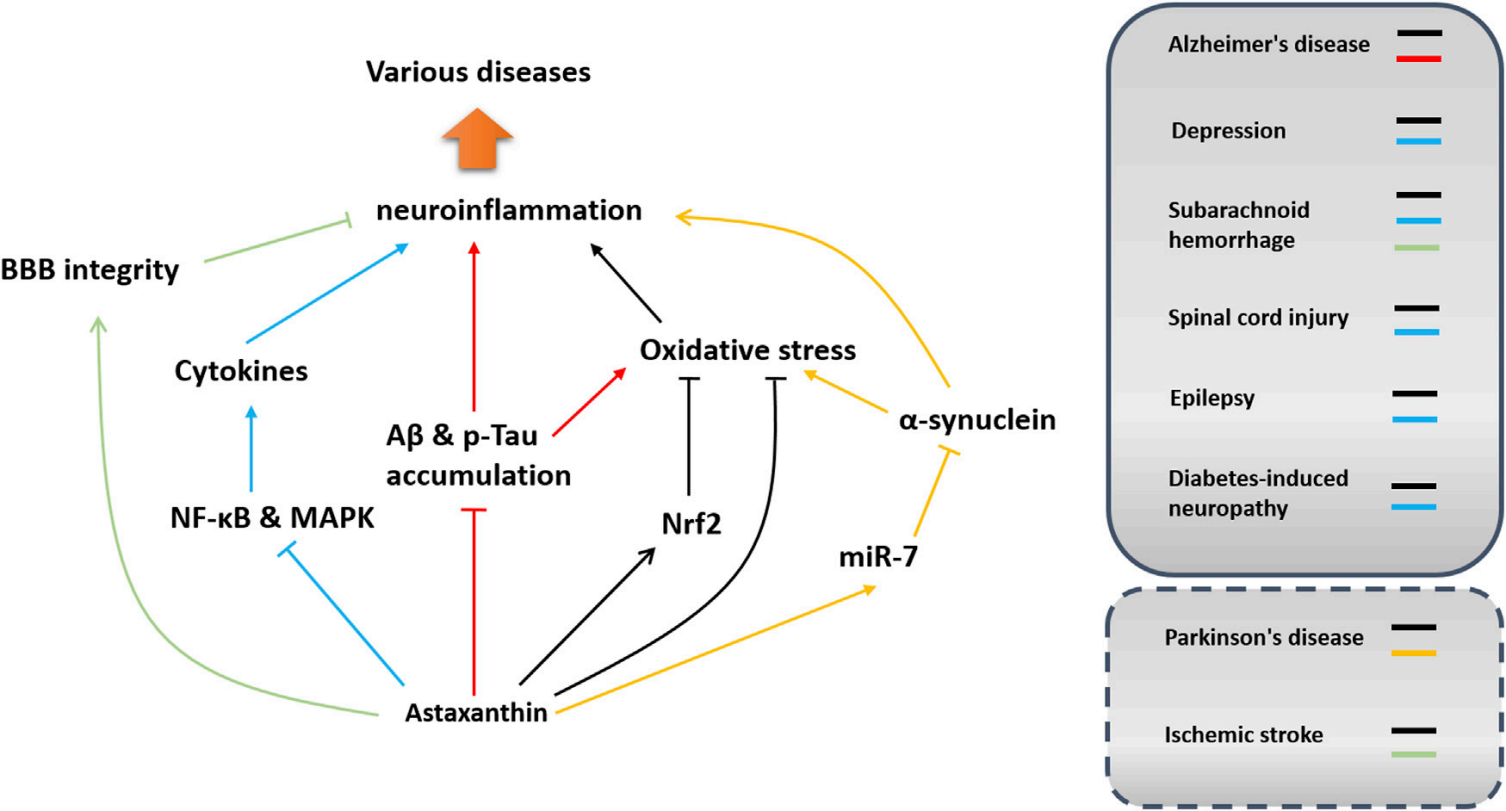
Implications and potential mechanisms of astaxanthin in neuroinflammation-associated disorders. Potential pathways involved in neuroinflammation modulation in different disease models are depicted in different colors. Arrows indicate acceleration or promotion, while lines with T-shaped ends represent inhibition or blocking. Dashed boxes indicate that the function of astaxanthin in modulating neuroinflammation has not been confirmed in these diseases.

Neuroinflammation is prevalent in neurodegenerative and neurodevelopmental diseases and metabolic neuropathy. Notably, astaxanthin has shown efficacy in modulating neuroinflammation in different disease models. Because astaxanthin has good biosafety and high bioavailability, its medical use, especially in modulating neuroinflammation, has always been a hot topic that warrants further investigation. In this section, the therapeutic benefits of astaxanthin against neurological disorders are summarized concerning neuroinflammation modulation. Its effects and potential mechanisms of action in inhibiting neuroinflammation in different diseases are shown in Figure 5 and Table 1.

**TABLE 1:** Effects and potential mechanisms of astaxanthin in modulating neuroinflammation.

Disease Model	Animal or Cell Line	Formulation, Dosage, and Treatment Time	Effects	Potential Target	References
Alzheimer’s disease	APP/PS1 double-transgenic mouse model	0.2% docosahexaenoic-acid-acylated astaxanthin diesters (DHA-AST) administered (p.o.) in AIN-93G diet for 60 days	Suppressed activation of microglia and astrocytes, inhibited inflammasome activation and attenuated proinflammation cytokine production	Unclear	Che et al. (2018)
	AD rat model induced by cerebral ventricle injection of Aβ (1–42)	0.5 mg/kg/day or 1 mg/kg/day astaxanthin was administered (p.o.) for 28 days beginning from the 8th day of cerebral ventricle Aβ(1–42) injection	Attenuated proinflammation cytokine production and oxidative stress in the hippocampus	Unclear	Rahman et al. (2019)
	AppNL-G-F transgenic mouse model	0.02% astaxanthin as free form (w/w) was administered in the diet for about 5 months	Attenuated oxidative stress and microglia accumulation in the hippocampus	Unclear	Hongo et al. (2020)
	Rat model induced by intraventricular infusion of ferrous amyloid buthionine (FAB)	1 ml/kg (body weight)/day astaxanthin administered in 0.5% DMSO in saline (i.p.) for 7 days	Suppressed activation of microglia and astrocytes	Nrf2	Chen et al. (2021a)
Depression	LPS-induced depressive-like mouse model	Pretreatment with 20,40, or 80 mg/kg trans-astaxanthin (p.o.) for 7 days	Attenuated proinflammation cytokine production in the hippocampus and prefrontal cortex	NF-κB	Jiang et al. (2016)
	Diabetes-related depressive-like mouse model	25 mg/kg/day astaxanthin in olive oil (p.o.) administered for 10 weeks	Suppressed astrocytes activation and attenuated proinflammation cytokine production	Unclear	Zhou et al. (2017)
Epilepsy	Status epilepticus rat mode	30 mg/kg/day astaxanthin in DMSO administered for 2 weeks	Suppressed microglia activation and attenuated proinflammation cytokine production	Unclear	Wang et al. (2020b)
	Status epilepticus rat mode	30 mg/kg astaxanthin in polyethylene glycol and tri-distilled water (1:1) was administered seven times (i.p.) in 14 days after establishing the model	Attenuated proinflammation cytokine production	Nrf2 and NF-κB	Deng et al. (2019)
Subarachnoid hemorrhage	Rat	25 or 75 mg/kg astaxanthin in olive oil (p.o.) administered 30 min after subarachnoid hemorrhage	Attenuated BBB disruption and proinflammation cytokine production	NF-κB	Zhang et al. (2014a)
Spinal cord injury	Rat	10 μl astaxanthin in 5% DMSO at a concentration of 0.2 mM injected (i.t.) 30 min after injury	Attenuated proinflammation cytokine production	MAPK	Fakhri et al. (2018)
Diabetes-induced neuropathy	Diabetic mouse model	25 mg/kg/day astaxanthin (p.o.) in olive oil for 7 days	Suppressed microglia activation and attenuated proinflammation cytokine production	NF-κB	Zhou et al. (2015)
General neuroinflammation	LPS-induced mouse model	20, 40, or 80 mg/kg astaxanthin in 0.5% sodium carboxy methyl cellulose administered (p.o.) for seven consecutive days before LPS injection	Attenuated proinflammation cytokine production	NF-κB	Jiang et al. (2016)
	LPS-induced mouse model	30 or 50 mg/kg/day astaxanthin in olive oil administered (p.o.) for 4 weeks	Attenuated proinflammation cytokine production	STAT3	Han et al. (2019)
	LPS-induced BV2 cell line model	5, 10, or 20 μM astaxanthin for 3 h before LPS addition	Attenuated LPS-induced neuroinflammation		
	LPS-induced mouse model	25 mg/kg/day astaxanthin emulsion administered intragastrically for 37 days	Attenuated proinflammation cytokine production	Unclear	Zhao et al. (2021)
	LPS-induced mouse model	40 mg/kg/day astaxanthin administered (p.o.) for 2 weeks	Suppressed microglia activation and attenuated proinflammation cytokine production	miR-31-5p and Notch	Zhou et al. (2021)
	LPS-induced BV2 cell line model	25 μM astaxanthin for 6 h	Attenuated proinflammation cytokine production		
	Kaliotoxin-induced mouse model	80 mg/kg astaxanthin administered (p.o.) twice at 1 and 5 h prior to kaliotoxin injection	Attenuated proinflammation cytokine production and BBB disruption	NF-κB	Sifi et al. (2016)
	Tobacco-induced mouse model	40 or 80 mg/kg astaxanthin in olive oil administered (p.o.) once per day for 10 days	Attenuated proinflammation cytokine production	MAPK	Yang et al. (2019)
	LPS-induced Rat microglia	10–500 μM astaxanthin dissolved in DMSO for 48 h	Attenuated proinflammation cytokine production	ATP-P2X7RSignal	Wang et al. (2020b)
	Lanthanum oxide nanoparticle-induced mouse model	60 mg/kg/day astaxanthin in olive oil administered intragastrically for 30 days	Attenuated proinflammation cytokine production	Nrf2	Yuan et al. (2020)
	LPS-induced BV2 cell line model	2–10 μM astaxanthin for 4 h	Attenuated proinflammation cytokine production	NF-κB	Wen et al. (2017)

### Alzheimer’s Disease

Chronic, aberrant neuroinflammation is a hallmark of different neurodegenerative diseases. Alzheimer’s disease (AD), a progressive neurodegenerative disorder characterized by memory loss and dementia, is always combined with neuroinflammation in the brain. Considering the beneficial effects of astaxanthin in modulating neuroinflammation, this carotenoid could be developed as a therapeutic agent for preventing or alleviating neurodegeneration. A randomized, double-blind clinical trial showed that daily supplementation with a combination of 3 mg astaxanthin and 5 mg sesamin for 6 weeks improved cognitive function in those with mild cognitive impairment (Ito et al., 2018).

AD is characterized by the aggregation of neurotoxic proteins, such as β-amyloid (Aβ) and hyperphosphorylated Tau (p-Tau), in the central nervous system, leading to chronic neuroinflammation by triggering microglial activation (Leng and Edison, 2021). Some studies have shown that astaxanthin (or astaxanthin-derived diesters) could reduce Aβ_42_ deposition and Tau phosphorylation, resulting in the suppression of neuroinflammation and enhancement of learning and memory in APP/SP1 transgenic mice, APP^NL−G-F^ mice, or Aβ-infused AD rat (Che et al., 2018; Rahman et al., 2019; Taksima et al., 2019; Hongo et al., 2020). Astaxanthin likely reduces Aβ generation and Tau phosphorylation by inhibiting GSK3β activity (Figure 2) (Rahman et al., 2019). GSK3 can phosphorylate Tau at more than 42 sites (Toral-Rios et al., 2020) and its activity strongly correlates with the number of neurofibrillary tangles in AD brains (Leroy et al., 2002). GSK3β can also enhance Aβ production by promoting *BACE1* transcription (Ly et al., 2013), which can partially explain the inhibitory effects of astaxanthin on BACE1 expression in AlCl_3_-induced AD-like rats (Hafez et al., 2021).

Moreover, impaired proteostasis caused by Aβ and p-Tau accumulation in the AD brain further contributes to oxidative stress, producing excessive reactive oxygen and nitrogen species. Oxidative stress has been detected in the early stages of AD, shown by a reduction in the levels of detoxifying enzymes, including superoxide dismutase (SOD), catalase (CAT), and glutathione peroxidase enzymes (GPx) (Cioanca et al., 2013; Hritcu et al., 2014). Astaxanthin can suppress oxidative stress by improving GPx activity, inhibiting lipid peroxidation, and reducing products of protein oxidation and superoxide anion in the cortex and the hippocampus of AD rats (Taksima et al., 2019). Therefore, astaxanthin can potentially suppress neuroinflammation by alleviating oxidative stress in the context of AD. Astaxanthin significantly elevated Nrf2 in an AD-like rat model (Hafez et al., 2021), consistent with its augmentative effect on Ho-1 enzyme expression (Wang et al., 2010; Wen et al., 2015). These data suggest that astaxanthin might also attenuate oxidation stress and subsequent neuroinflammation by regulating the Nrf2 pathway in the context of AD. Astaxanthin was also reported to decrease neuroinflammation, restore choline acetyltransferase positive fibers, increase the spine numbers of pyramidal neurons in the hippocampal CA1 region, and ameliorate the behavioral deficits in a ferrous amyloid buthionine (FAB)-infused sporadic AD rat model (Chen MH. et al., 2021). Astaxanthin likely modulates cholinergic decline by increasing the expression of nerve growth factor (NGF), which could prevent the degeneration of cholinergic neurons (Counts and Mufson, 2005; Nai et al., 2018). Thus, astaxanthin represents a potential therapeutic agent for slowing AD progression by inhibiting neuroinflammation.

In the AD brain, increased levels of pro-inflammatory cytokines (e.g., TNF-α, IL-1, and IL-12) may contribute to aberrant neuroinflammation and neurological impairment by triggering sustained microglial activation (Ahmad et al., 2022). Impaired BBB integrity in AD facilitates the infiltration of immune cells and cytokines from the peripheral blood that function as adverse factors or accomplices with resident immune cells to trigger neuroinflammation (Zenaro et al., 2017). Astaxanthin can reduce the release of pro-inflammatory factors in the BV2 cell line by regulating the NF-κB and MAPK pathways and can also protect BBB integrity and inhibit peripheral inflammation. However, whether astaxanthin can ameliorate AD neuropathy by modulating neuroinflammation through these mechanisms remains unknown.

### Parkinson’s Disease

Neuroinflammatory processes are also involved in the pathogenesis of Parkinson’s disease (PD). Increased pro-inflammatory cytokines levels in the brain, cerebrospinal fluid (CSF), and blood have been found postmortem in PD patients (Nagatsu et al., 2000). Abnormal neuroinflammation has also been observed in PD models induced by 6-hydroxydopamine, MPTP (1-methyl-4-phenyl-1,2,3,6-tetrahydropyridine), and α-synuclein (Hirsch and Hunot, 2009). Chronic, prolonged inflammation in microglia, which induces the death and dysfunction of neighboring dopaminergic neurons, is a crucial mechanism underlying the pathogenesis of PD. Hence, immune-based neuroprotection is widely considered an effective approach in PD therapy (Hirsch and Standaert, 2021). Astaxanthin has shown promising therapeutic effects in PD, which have been discussed in other reviews (Galasso et al., 2018; Park HA. et al., 2020; Bahbah et al., 2021). Whether astaxanthin can modulate neuroinflammation in the context of PD remains elusive. Focusing specifically on modulating neuroinflammation, astaxanthin deserves more in-depth investigation for PD treatment. Aggregated α-synuclein can stimulate the expression of pro-inflammatory cytokines (e.g., IL-1β, TNF-α, and IFN-γ) through activation of the NF-κB pathway in microglia (Reynolds et al., 2008). Astaxanthin significantly reduces α-synuclein expression and apoptosis in SH-SY5Y cells treated with 1-methyl-4-phenylpyridinium (MPP+), which resembles PD by initiating neuron death via a miR-7-dependent pathway (Shen et al., 2021). Because astaxanthin can attenuate NF-κB activation (Figure 3), it may act by suppressing neuroinflammation induced by α-synuclein in PD. Similarly, astaxanthin can attenuate PD progression by reducing apoptosis in dopamine neurons through the P38 MAPK pathway (Wang CC. et al., 2020) while also triggering the production of pro-inflammatory cytokines (Figure 3).

Oxidative stress in the PD brain has been observed in postmortem human samples and animals and is another risk factor leading to the dysfunction of dopaminergic neurons (Gilgun-Sherki et al., 2001; Gaki and Papavassiliou, 2014). Mitochondrial dysfunction-induced oxidative stress can trigger neuroinflammation-associated dopaminergic neuronal cell death and parkinsonism (Shulman et al., 2011). Considering astaxanthin can regulate mitochondrial function that prevents the excessive production of ROS, it could be used as a protective agent to suppress neuroinflammation-associated parkinsonism. Astaxanthin has exhibited an anti-oxidative effect in MPP-induced PC12 cells through the HO-1/NOX2 and Sp1/NR1 pathways (Ye et al., 2012; Ye et al., 2013) and an anti-apoptosis effect in 6-hydroxydopamine-induced SH-SY5Y cells via a mitochondria-targeted protective mechanism (Ikeda et al., 2008; Liu X. et al., 2009).

### Depression

Dysfunctional immune and endocrine systems are two contributors to depression. Neuroinflammation in the brain is associated with the pathophysiology of depression, although the mechanism remains unsolved (Kim et al., 2016). The P2x7-Nlrp3 inflammasome cascade acts as a key mechanism underlying depression. The NF-κB and MAPK pathways, through their control of pro-inflammatory cytokine production and NLRP3 inflammasome activation, are recognized as two significant mechanisms in the pathogenesis of depression (Troubat et al., 2021). Trans-astaxanthin can rescue LPS-induced depressive-like behavior by antagonizing neuroinflammation in an NF-κB-dependent manner (Jiang et al., 2016). Moreover, astaxanthin has anti-depressant effects on diabetic mice by inhibiting inflammation. Astaxanthin can significantly reduce the number of GFAP-positive cells (mostly astrocytes) in the hippocampus and hypothalamus and downregulate the expression of IL-6, IL-1β and COX-2 in the hippocampus of depressive mice (Zhou et al., 2017). Taken together, these data suggest that astaxanthin could be an effective therapeutic agent for depression by targeting the associated inflammation.

Depression is also accompanied by aberrant oxidative stress in the brain, manifested as increased lipid peroxidation and free radicals, an abnormal antioxidant system, oxidative damage,e and pro-inflammatory cytokine overproduction (Liu et al., 2015; Vavakova et al., 2015). Impaired activation of the Nrf2-dependent antioxidant system can lead to stress-induced vulnerability to depression. Several drugs targeting the Nrf2 pathway (e.g., melatonin and edaravone) have beneficial effects against neuroinflammation and depressive-like behaviors (Maes et al., 2012; Arioz et al., 2019; Dang et al., 2022). Considering astaxanthin as a potential neuroinflammation modulator counteracting oxidative stress via the Nrf2 pathway, it may also be effective in treating depression.

### Cardiac-Cerebral Vascular Diseases

Subarachnoid hemorrhage (SAH) caused by traumatic or non-traumatic cerebral angiorrhexis is associated with profound systemic complications, leading to high mortality rates and long-term neurological disabilities (Macdonald, 2014; Macdonald and Schweizer, 2017). Systemic immune responses always occur after SAH, which is commonly manifested by high levels of pro-inflammatory cytokines (IL-1, IL-6, and TNF-α) in peripheral blood and brain (Jung et al., 2013). Moreover, resident immune responses mediated by microglia activation in the brain can cause secondary brain damage after SAH (Heinz et al., 2021). Therefore immunosuppressive treatment is effective and fundamental to improving SAH prognosis. Astaxanthin antagonizes neuronal apoptosis by counteracting the neuroinflammatory responses after subarachnoid hemorrhage (SAH). The neuroprotective effect of astaxanthin in SAH is mediated by regulating mitochondrial function through the phosphorylation-dependent inactivation of BCL2, a crucial agonist of cell death. Astaxanthin may regulate BCL2 phosphorylation through the PI3K/Akt pathway (Zhang et al., 2014b). Moreover, astaxanthin could attenuate neuroinflammation by rescuing BBB disruption and inhibiting NF-κB-dependent expression of inflammatory cytokines in a SAH rat model (Zhang et al., 2014a).

Ischemic stroke caused by cerebral infarction is a common cerebrovascular disease and a striking cause of death and severe disability worldwide (Feske, 2021). Pro-inflammatory signals can rapidly activate resident immune cells in the brain in response to ischemic stroke and promote the infiltration of a wide range of peripheral inflammatory cells into the ischemic region, exacerbating brain damage (Jayaraj et al., 2019). Neuroinflammation, which can be both beneficial and detrimental, has become a widely studied target for therapeutic intervention for ischemic stroke. Notably, astaxanthin has shown a neuroprotective effect after ischemic stroke by attenuating oxidative stress. For example, astaxanthin could reduce brain injury in a rat ischemic stroke model by decreasing oxidative stress and inhibiting glutamate overflow (Shen et al., 2009). In other studies, astaxanthin attenuated oxidative stress and promoted axon regeneration and reconnection after ischemic stroke (Lee et al., 2010; Wang et al., 2019). In an oxygen and glucose deprivation (OGD) model, astaxanthin treatment protected cultured SH-SY5Y cells against OGD-induced cytotoxicity by modulating oxidative stress (Zhang J. et al., 2020). Impaired BBB permeability after ischemic stroke is an underlying cause of the invasion of peripheral inflammatory cells into the brain (Jayaraj et al., 2019). RNS are reactive molecules that trigger BBB disruption following cerebral ischemia-reperfusion injury (Chen et al., 2013). They are recognized as important therapeutic targets for identifying drug candidates for attenuating brain injury (Chen H. et al., 2018; Chen HS. et al., 2018; Feng et al., 2018; Chen et al., 2020). Astaxanthin can quench RNS, such as peroxynitrite (Rodrigues et al., 2012) and nitrogen monoxide (Khan et al., 2010), which might reduce RNS-induced BBB impairment under some pathological conditions. The protective effect of astaxanthin on BBB integrity provides another mechanism for anti-inflammation in ischemic stroke; however, the mechanism requires further elucidation. The available evidence indicates that astaxanthin could be used as a potential neuroinflammation modulator in response to ischemic stroke-induced brain damage.

The interaction between neuroinflammation and cardiovascular disease has recently become a research focus. Neuroinflammation is both a cause and a consequence of cardiovascular disease (Richards et al., 2022). Neuroinflammation has been implicated in hypertension (in animal models and humans) through multiple mechanisms (Mowry and Biancardi, 2019; Sharma et al., 2019). It is particularly apparent in the hypothalamic paraventricular nucleus (PVN) of hypertensive rodents (Sklerov et al., 2019). Moreover, increased expression of pro-inflammatory cytokines has been reported in cardio-regulatory brain regions in hypertensive animals (Shi et al., 2010b; Qi et al., 2016) and angiotensin II-induced hypertension was found to trigger microglial activation predominantly in the PVN (Li Y. et al., 2020). In contrast, the administration of anti-inflammatory cytokines (e.g., IL-10) and minocycline induced an antihypertensive response by alleviating microglial activation (Shi et al., 2010a).

Astaxanthin is recognized as a potential therapeutic agent against cardiovascular disease. Disodium disuccinate astaxanthin protected the myocardium in a myocardial ischemia-reperfusion model by reducing inflammation and myocardial apoptosis (Gross and Lockwood, 2005; Lauver et al., 2005; Gross et al., 2006). In addition, astaxanthin improved arterial hypertension by decreasing the production of superoxide anions in rodents (Monroy-Ruiz et al., 2011). Moreover, astaxanthin may be protective against atherosclerotic cardiovascular disease by reducing oxidative stress and inflammation and improving glucose metabolism (Fassett and Coombes, 2011; Kishimoto et al., 2016). Astaxanthin is a likely candidate for treating cardiovascular disease, considering its cardiovascular protective effects and its therapeutic effects against neuroinflammation.

### Spinal Cord injury

Spinal cord injury (SCI) is a devastating condition associated with impaired motor ability and long-term comorbidity. Neural restoration in the spinal cord remains the most effective treatment for SCI, while aberrant neuroinflammation contributes to a poor prognosis (Brockie et al., 2021). Astaxanthin can decrease the production of TNF-α by modulating the phosphorylation of MAPK p38 (threonine 180 and tyrosine 182) and improve sensory and motor function following rat spinal cord injury (Fakhri et al., 2018), indicating that this agent can inhibit inflammatory reactions in the secondary phase of SCI. Thus, it represents a promising candidate for enhancing functional recovery after SCI. Inhibiting oxidative stress and Nrf2 activation may also promote functional recovery after SCI (Jin et al., 2021). Therefore, astaxanthin treatment might improve neural restoration by counteracting oxidative stress-induced neuroinflammation.

### Epilepsy

Alleviating neuroinflammation can also reduce cellular damage caused by epilepsy (Jayaraj et al., 2019; Parsons et al., 2022). Neuroinflammation also acts as a trigger for epilepsy. Pro-inflammatory cytokines (e.g., IL-1β, IL-6, and TNF-α) can induce this disorder (Vezzani et al., 2016). Indeed, increased levels of these cytokines have been found in the cerebrospinal fluid (CSF) and blood serum of patients with epilepsy (Dupuis and Auvin, 2015). Thus, astaxanthin is a potential agent for improving the prognosis of epilepsy patients by inhibiting the neuroinflammation-associated injury following a seizure (Lu et al., 2015; Deng et al., 2019).

### Diabetes-Induced Neuropathy

Diabetes-induced neurological complications, such as vascular pathogenesis in the brain, impaired neuronal regeneration, neurodegeneration, and peripheral neuropathy, are major obstacles to improving the quality of life of diabetes patients (Asslih et al., 2021). Aberrant neuroinflammation is an important mechanism triggering these complications. Therefore, inhibitors of neuroinflammation (e.g., astaxanthin) could serve as auxiliary supplements or drugs for alleviating diabetic neuropathy (Asslih et al., 2021). Astaxanthin can prevent diabetic nephropathy and renal cell damage by reducing oxidative stress and inflammation (Naito et al., 2004; Manabe et al., 2008; Kim et al., 2009). In addition, insulin sensitivity is enhanced after feeding mice astaxanthin (Bhuvaneswari et al., 2010). Moreover, it has provided a significant beneficial effect in the treatment of diabetes. For example, astaxanthin can reduce oxidative stress-induced hyperglycemia in pancreatic β-cells, improving serum insulin and glucose levels (Uchiyama et al., 2002; Nakano et al., 2008). However, whether the improved blood glucose levels generated by astaxanthin can inhibit neuroinflammation remains unclear.

## Challenges and Prospects for the Medical Application of Astaxanthin

### Source, Safety, and Bioavailability

Astaxanthin is commercially used as both a food additive and supplement. Synthetic astaxanthin is mostly consumed as a food additive for animals, while only natural astaxanthin can be used as a food ingredient for humans. In 1987, the United States Food and Drug Administration (FDA) approved synthetic astaxanthin produced by Roche as a food additive in the aquaculture industry, while natural astaxanthin was approved as a nutraceutical for humans in 1999. The chemical process by which astaxanthin is synthesized can inevitably generate detrimental by-products, which reduce its biosafety and bioactivity. Natural astaxanthin can be sourced from microalgae, yeast, shrimp, krill, and plankton; however, it is primarily commercially produced from *Phaffia rhodozyma* and *H. pluvialis* due to their advantages of astaxanthin content, growth rate, and cost of cultivation. *H. pluvialis*, a green freshwater microalga, is widely recognized as the optimal natural microbial factory for astaxanthin production due to its high carotenoid content and easy extraction method. *H. pluvialis* can synthesize and accumulate astaxanthin up to 4% of total cellular dry weight. The high protein content of microalgae is another reason for the development of *H. pluvialis* as a source of astaxanthin production (Hu, 2019). There are currently commercial producers of astaxanthin-rich *H. pluvialis* worldwide, with a large output capacity. Optimizing the cultivation strategy and developing an effective extraction method has reduced the cost of industrial production, promoting natural astaxanthin, which is more accepted for public consumption in various countries. Moreover, the increasing awareness of the multifunctional benefits of astaxanthin should induce rapid growth in this market. According to Grand View Research, a market research and consulting company in the US, the compound annual growth rate of the astaxanthin market is expected to increase by 19.3% from 2021 to 2028.

The safety of astaxanthin for use in humans is well documented. The FDA has approved astaxanthin sourced from *H. pluvialis* as a food ingredient since 2010 (GRAS [Generally Recognized as Safe] No. 294 and No. 580). In Europe, astaxanthin from *H. pluvialis* is authorized by the European Food Safety Authority Commission as a food supplement at levels up to 8 mg/day for adults (EFSA Panel on Nutrition Novel Foods and Food Allergens et al., 2020). In 2010, astaxanthin-rich *H. pluvialis* was also listed as an edible strain by the Ministry of Public Health of China. Taking these developments together, astaxanthin is generally accepted as safe for food and is in growing demand. Thus, astaxanthin products have great potential to be integrated into everyday life, bringing benefits to public health.

Astaxanthin is a fat-soluble compound incorporated into micelles with lipids for absorption by mucosal cells in the intestinum tenue. In the form of chylomicrons, it is transported into the liver via the lymphatic system and subsequently transported by lipoproteins to different organs and tissues via the circulation (Parker, 1996; Coral-Hinostroza et al., 2004). The form of astaxanthin can affect its absorption efficiency. According to clinical research, the maximum plasma level of astaxanthin can reach up to 1.3 ± 0.1 mg/L with a plasma elimination half-life of 21 ± 11 h after a single dose of 100 mg of free astaxanthin (Osterlie et al., 2000). In contrast, ingestion of 100 mg of astaxanthin diesters results in a maximum plasma astaxanthin level of 0.28 ± 0.12 mg/L with a plasma elimination half-life of 52 ± 40 h, indicating the complicated absorption of astaxanthin due to the additional hydrolysis of astaxanthin esters (Coral-Hinostroza et al., 2004). The bioavailability of astaxanthin is also influenced by concomitant diet and lifestyle. Its consumption in combination with oil or an oil-based formulation can enhance the absorption of astaxanthin (Mercke Odeberg et al., 2003), while smoking can reduce its bioavailability by decreasing its half-life (Okada et al., 2009).

### Unresolved Issues in the Medical Application of Astaxanthin

The development of astaxanthin as an effective modulator to relieve dysregulated neuroinflammation may be an effective neuroprotective strategy; however, challenges remain. First, neuroinflammation functions as a double-edged sword for maintaining the homeostasis of the nervous system. Acute neuroinflammation is beneficial in rebuilding balanced metabolism by clearing cellular debris and pathogens, but aberrantly prolonged or chronic neuroinflammation may be harmful. Hence, it may be an oversimplification to use astaxanthin as an inhibitor of neuroinflammation in all situations. Furthermore, animal experiments and clinical research have indicated that the long-term consumption of high-dose astaxanthin is associated with its medical efficacy. Therefore, the likelihood of incidental adverse effects, such as pigmentation and allergies (Nutrition and Allergies EFSA Panel on Dietetic Products, 2014), need to be studied. Moreover, the role of astaxanthin in abrogating neuroinflammation is complex, and side effects due to the targeting of the NF-κB, Nrf2, and MAPK pathways remain a concern for any potential clinical applications (Sporn and Liby, 2012; Ramadass et al., 2020; Zang et al., 2020; Moreira et al., 2021). Therefore, the molecular mechanisms of astaxanthin in these pathways require further investigation. Thus far, astaxanthin has only been shown to directly interact with p38 MAPK (Yang et al., 2019), while other direct targets for astaxanthin in different pathways remain elusive.

In summary, astaxanthin is currently only used as a food ingredient. The potential efficacy of astaxanthin as a drug that modulates neuroinflammation requires further elucidation, although its neuroprotective effects are well-documented. First, there is a need to explore the specific target(s) of astaxanthin in different pathways to further understand the underlying mechanisms of its modulation of neuroinflammation. In addition, the combination of astaxanthin with other existing chemicals may more effectively counteract neuroinflammation (Polotow et al., 2015; Qiao et al., 2017; Ito et al., 2018). Moreover, improved formulations (e.g., microencapsulation) or modified forms (e.g., docosahexaenoic-acid-acylated astaxanthin) may enhance the bioavailability and bioactivity of this agent (Yang et al., 2021; Zhao et al., 2021).

Some issues with the potential adverse effects of astaxanthin may provide more misgivings during its medical application, although they have not been reported in animal studies or clinical research using a reasonable dose range according to FDA GRAS notices. Some ingredients (e.g., sunflower or krill oil) may be used in astaxanthin production. Thus, allergenicity may remain uncertain, especially for immunocompromised individuals. In addition, astaxanthin can also induce cytochrome P450 enzymes in rats (Ohno et al., 2011) and primary human hepatocytes (Kistler et al., 2002). However, an FDA GRAS panel has concluded that astaxanthin at human exposure levels is unlikely to affect cytochrome P450 enzymes. Pigmentation in human tissues has also been raised as a concern (Petri and Lundebye, 2007); however, the FDA GRAS panel has also clarified this issue by concluding that the proposed astaxanthin dose levels do not raise safety concerns for pigmentation in humans.

## Conclusion

Neuroinflammation functions as a defense mechanism to protect the central nervous system from different insults; however, it is also a pathological hallmark of numerous neurological and neurodegenerative diseases. Astaxanthin, a natural carotenoid with marked antioxidant capacity, suppresses neuroinflammation and is thus neuroprotective. First and foremost, astaxanthin can effectively counteract the oxidative stress-induced cell injury and death known to trigger neuroinflammation, in part, by inhibiting the production of pro-inflammatory cytokines via the NF-κB and MAPK pathways. It can also potentially modulate neuroinflammation in the brain by maintaining the integrity of the BBB and alleviating peripheral inflammation. To date, astaxanthin has been developed as a commercial food ingredient with well-documented biosafety, numerous bioactivities, and a reasonable price. At the same time, it also exhibits abundant therapeutic benefits for modulating neuroinflammation. Although several issues concerning its medical efficacy and mechanisms for treating neuroinflammation-associated diseases require further elucidation, astaxanthin remains a prospective medicinal component for the modulation of neuroinflammation.
